# SLPI controls neutrophil migration abilities and impacts neutrophil skin infiltration in experimental psoriasis

**DOI:** 10.1007/s00018-025-05606-y

**Published:** 2025-02-10

**Authors:** Patrycja Kwiecinska, Michal Santocki, Joanna Skrzeczynska-Moncznik, Ivan Sinkevich, Katarzyna Piwowarczyk, Pawel Majewski, Beata Grygier, Monika Majchrzak-Gorecka, Jaroslaw Czyz, Elzbieta Kolaczkowska, Joanna Cichy

**Affiliations:** 1 Department of Immunology, Faculty of Biochemistry, Biophysics and Biotechnology, Jagiellonian University, Gronostajowa 7 St, Krakow, 31-571 Poland; 2 Present address: Laboratory of Stem Cell Biology, Faculty of Biochemistry, Biophysics and Biotechnology, Jagiellonian University, Krakow, Poland; 3 Laboratory of Experimental Hematology, Institute of Zoology and Biomedical Research, Jagiellonian University, Krakow, Poland; 4 Faculty of Biochemistry, Biophysics and Biotechnology, The Doctoral School of Exact and Natural Sciences of the Jagiellonian University, Krakow, Poland; 5 Department of Cell Biology, Faculty of Biochemistry, Biophysics and Biotechnology, Jagiellonian University, Krakow, Poland; 6 Present address: Department of Experimental Neuroendocrinology, Maj Institute of Pharmacology, Polish Academy of Science, Krakow, Poland; 7 Present address, Krakow: Ardigen, Krakow Poland

**Keywords:** Dermis, Chronic inflammation, Inhibitors of serine proteases, Innate immunity, Intravital microscopy, Neutrophil elastase

## Abstract

Skin infiltration by neutrophils is a hallmark of the chronic inflammatory skin disease psoriasis, yet the mechanisms underlying neutrophil recruitment and positioning in chronically inflamed skin remain poorly understood. In this study, we demonstrate the significant impact of a total genetic deficiency of secretory leukocyte protease inhibitor (SLPI) on neutrophil migration in mouse skin. Without SLPI, neutrophils displayed an unconventional migratory pattern, characterized by altered interactions with vessel walls and reduced efficiency in extravasating from blood vessels into skin tissue during the early stages of experimental psoriasis. This was associated with changes in tissue motility, positioning neutrophils farther from the skin entry vessels and closer to the skin surface. Neutrophil diapedesis was partially dependent on SLPI within the neutrophils themselves. The impact of SLPI on neutrophil movement was further supported by the increased migration of human neutrophils in the presence of neutrophil-penetrant recombinant SLPI. Additionally, our data suggest that neutrophils with varying capacities for vessel wall interaction are released from the bone marrow into circulation in an SLPI-dependent manner. These findings establish a role for SLPI in regulating the spatiotemporal infiltration of neutrophils into the skin in psoriasis, highlighting its relevance to psoriasis pathophysiology.

**Supplementary Information:**

The online version contains supplementary material available at 10.1007/s00018-025-05606-y.

## Introduction

Neutrophils have a leading role in innate defenses against microbes, but their contribution to chronic inflammation remains less well understood. This ambiguity is exemplified in psoriasis, a chronic inflammatory skin disorder characterized by early neutrophil infiltration into developing cutaneous lesions [1]. The accumulation of neutrophils in the dermis and epidermis is a histological hallmark of psoriasis, yet specific role of neutrophils in the disease remains unclear. Neutrophils have been implicated in the pathogenesis of psoriasis by facilitating abnormal proliferation and differentiation of keratinocytes, enhancing vascular permeability, and promoting inflammation and the expansion of memory Th17 cells [2–8]. However, neutrophils are also involved in the removal of damaged cells and cellular debris [9], potentially indicating a protective role in the skin during psoriasis. The migration of neutrophils to chronically inflamed skin is essential for their pathogenic or protective functions. Therefore, understanding the mechanisms underlying neutrophil accumulation in psoriasis could provide crucial insights into the involvement of these cells in the disorder.

The egress of neutrophils from the vasculature and their mobilization to sites of inflammation is intricately controlled by a cascade of interactions with activated endothelial cells. This cascade involves several steps: initial slowing down of neutrophils on the endothelium (tethering, which is the loose binding of free-flowing neutrophils to endothelial cells) and rolling, followed by firm attachment to endothelial cells, crawling (as neutrophil search for a site for transmigration), and finally, transmigration (diapedesis) through the endothelial lining into adjacent connective tissue and further upwards to the epidermis and the skin surface [10–12]. Of note, neutrophils in psoriatic skin lesions are often observed in perivascular areas around dilated vessels, contributing to vascular remodeling and dilation [2].

Neutrophils express specific surface adhesion molecules that facilitate rapid trafficking. The levels of expression, distribution across cells, and functional activity of these molecules are crucial for delivering neutrophils to inflamed tissues [10, 11]. In addition to adhesion molecules, neutrophil tissue mobilization depends on soluble and intracellular trafficking signals. Neutrophils are a rich source of proteolytic enzymes, such as the serine protease neutrophil elastase (NE). NE has been implicated in neutrophil migration, either through the intracellular processing of cytoskeletal components in neutrophils or via the cleavage of junctional adhesion molecule -C (JAM-C) on endothelial cells [13, 14]. Notably, neutrophils also abundantly express inhibitors of these enzymes, including secretory leukocyte proteinase inhibitor (SLPI) [8, 15]. SLPI is regarded as key intracellular inhibitor of NE in neutrophils [16]. Both circulating human neutrophils and those recruited to psoriatic skin lesions contain high levels of SLPI (9). Despite these observations, the role of SLPI in neutrophils and its involvement in chronic inflammatory disorders remain poorly understood.

Given SLPI’s antiprotease activity against NE and other serine proteases, such as cathepsin G (CatG), we hypothesized that SLPI controls migration of neutrophils to sites of chronic inflammation. Using an experimental model of psoriasis, we demonstrate that the mobilization of neutrophils to diseased skin is significantly delayed in the absence of this inhibitor. Mechanistically, we show that SLPI affects neutrophil recruitment to psoriatic skin at the level of neutrophil extravasation from the blood and within the skin tissue, and likely regulates neutrophil release from the bone marrow into the bloodstream. The observed alterations in skin infiltration were at least partially dependent on the intrinsic action of SLPI in neutrophils. Furthermore, human neutrophils treated with recombinant SLPI exhibited enhanced migratory capacity.

Collectively, these findings identify SLPI as an important regulator of neutrophil recruitment to psoriatic skin, acting at multiple levels to influence the efficiency and pattern of neutrophil migration and potentially impacting the course of inflammation in psoriasis.

## Materials and methods

### Mouse models

All animal procedures and experiments were performed in accordance with national and European legislation, following approval by the 2nd Local Institutional Animal Care and Use Committee in Krakow (approval numbers 298/2017 and 61/2023). SLPI KO and WT mice were generously donated by Dr. Sharon M. Wahl [17, 18]. C57BL/6 mice were obtained from The Jackson Laboratory and housed under pathogen-free conditions in the animal facility at the Faculty of Biochemistry, Biophysics, and Biotechnology of the Jagiellonian University in Krakow. Littermate 10–12 weeks-old males were used for all experiments. A psoriasis model was induced using imiquimod (IMQ) [19]. Mice were treated twice daily for up to 6 days with 5 mg of Aldara (5% imiquimod cream) (Meda AB) applied to both sides of the depilated ear. For intravital imaging, mice were treated with IMQ for 2 days. Blood samples were collected into tubes containing 10 mM EDTA (Sarstedt). Bone marrow cells were isolated from femurs and tibias by centrifugation at 10,000 × g [20]. Ear biopsies were cut into small pieces and incubated with a 2.5 mg/mL Collagenase D (Roche Diagnostics) solution at 37 °C for 50 min. Single-cell suspensions from the ears were prepared by mashing the tissue through 40-µm cell strainers. Red blood cells were eliminated using RBC Lysis Buffer (eBioscience). The remaining cells were resuspended in RPMI 1640 medium (Biowest) supplemented with 2% heat-inactivated FBS (Gibco) and immediately stained for flow cytometry analysis.

### Human samples

All human studies were performed in accordance with guidelines established by the Jagiellonian University Institutional Bioethics Committee under approved protocols (#87/B/2014; 1072.6120.30.2020) and adhered to the Declaration of Helsinki. Human blood was collected from healthy individuals who were fully informed and had consented.

### Flow cytometry

Single-cell suspensions were stained for viability assessment (Zombie Aqua Fixable Viability Kit; BioLegend) and then unspecific antibody-binding sites were blocked with anti-CD16/CD32 antibodies (Fc block; eBioscience) followed by staining with directly conjugated antibodies: CD45.2-APC/Cy7 (BioLegend), CD11b-eFluor450 (eBioscience), Ly6G-APC (BioLegend) or c-Kit-PE/Cy7 (BioLegend), Lin-AF700 (BioLegend), c-Kit-APC/eF780 (eBioscience), CD34-FITC (eBioscience), Ly6G-BV711 (BD Biosciences). Data were acquired on a BD LSRII (BD Biosciences) and were analyzed using FCS Express (De Novo Software).

Singlets were selected on the basis of FCS-A vs. FCS-H. Dead cells were routinely excluded from the analysis. The frequencies of specific cell types were calculated as the percentage of CD45^+^ cells. Neutrophils were defined as live, CD45^+^, CD11b^+^ and Ly6G^+^ cells. Neutrophil progenitors were defined according to the gating strategy described previously [21]. The lineage antibody cocktail included anti-CD3 (clone 17A2), anti-Ly-6G/Ly-6 C (clone RB6-8C5), anti-CD11b (clone M1/70), anti-B220 (clone RA3-6B2) and anti-TER-119 (clone Ter-119) antibodies (BioLegend). c-Kit and CD34 were used to exclude CD34 – c-Kit^+^ stem/progenitor cells and FSC/SSC gating excluded eosinophils (SSC^high^). The differential expression levels of c-kit and Ly6G were used to define the developmental stages of neutrophils. “Fluorescence minus one” (FMO) controls were routinely used to set the thresholds for positive/negative events.

### Adoptive transfer

Bone marrow neutrophils were isolated by density gradient centrifugation. Diluted cell samples were layered on top of density gradient separation solutions (Pancoll human for Granulocytes 1.119 g/ml, Pancoll human 1.077 g/ml, and blood in a volume ratio of 1:1:2) (PAN Biotech). After centrifugation and washing, the remaining red blood cells in the granulocyte fraction were lysed using pyrogen-free water. The cells were then resuspended in RPMI 1640 medium (Biowest) supplemented with 10% heat-inactivated FBS (Gibco). The purity of the isolated cells was examined by flow cytometry based on CD45, CD11b, and Ly6G immunoreactivity, resulting in neutrophils with 64–80% purity.

The isolated neutrophils were labeled with CellTracker dyes (final concentration of 1 µM) for 15 min. at 37 °C and then washed twice with PBS (PAN Biotech) supplemented with bovine serum albumin (Sigma-Aldrich). CellTracker Red (CMTPX, Invitrogen) and CellTracker Green (CMFDA, Invitrogen) were used to differentially label neutrophils from WT and SLPI KO mice, respectively. Labeled neutrophils were resuspended in PBS (PAN Biotech) at a density of 1 × 10^8^/ml, and 100 µl of cell suspension was injected intravenously (i.v.) via the jugular vein cannula 30 min. before intravital microscopy imaging. Each time, 1 × 10^7^ neutrophils (mixed WT and SLPI-deficient neutrophils at a 1:1 ratio) were transferred per mouse.

### Preparation of the mouse ear skin for intravital microscopy (IVM)

Mice were anesthetized with a mixture of ketamine hydrochloride (200 mg/kg; Biowet Puławy) and xylazine hydrochloride (10 mg/kg; Dechra) administered intraperitoneally. After anesthesia, cannulation of the right jugular vein was performed to maintain anesthesia and for the administration of antibodies or cells [22]. Preparation of the ear skin for intravital imaging was performed immediately after the injection of antibodies. Briefly, the mouse was moved to a custom-made Plexiglas board designed for microscopic imaging. The mouse was placed on its back, and the ear to be imaged was positioned on a stack of eight microscope slides (76 × 26 mm, 1 mm thick) taped together with surgical tape, with the ventral side up. Using a diamond glass-cutting knife, a coverslip was cut to the desired size and placed on the ventral side of the ear, with saline immediately applied underneath the coverslip.

### Spinning disk confocal IVM

The mouse ear was visualized using a ZEISS Axio Imager.M2 upright microscope equipped with a metal halide light source (AMH-200-F6S; Andor, Oxford Instruments), a motorized 6-position excitation filter wheel, and a laser-free confocal spinning-disk device (DSD2; Andor, Oxford Instruments). The microscope utilized ZEISS EC Plan-NEOFLUAR 10×/0.3 and/or ZEISS EC Plan-NEOFLUAR 20×/0.5 air objectives. Four excitation filters were used: DAPI (390/40 nm), GFP (482/18 nm), RFP (561/14 nm), and Cy5 (640/14 nm), each visualized with the appropriate emission filters: DAPI (452/45 nm, exposure time 500 ms), GFP (525/45 nm, exposure time 500 ms), RFP (609/54 nm, exposure time 500 ms), and Cy5 (676/29 nm, exposure time 250 ms). Fluorescence detection was performed using a 5.5-megapixel sCMOS camera (Zyla 5.5; Andor, Oxford Instruments).

The iQ 3.6.1 acquisition software (Andor, Oxford Instruments) was used to control the microscope. A series of optical cross-sections (z-stacks) through the mouse ear skin was performed with a fully motorized microscope stage (Scan 130 × 85, ball screw pitch 2 mm; Märzhäuser Wetzlar). The z-step was set to 1 μm, and approximately 100 z-planes were captured, either in a single run or as a time-lapse acquisition over 10 min., with intervals of 750 ms, resulting in a total of 800 frames.

### Imaging of neutrophils and blood flow with IVM

Imaging of neutrophils and platelets (to indirectly visualize blood flow) in the ear skin tissue and blood vessels was performed by staining the cells with appropriate fluorochrome-conjugated monoclonal antibodies. Neutrophils were visualized by injecting Ly6G-AF647 antibodies (1 µg per mouse; BioLegend) and Ly6G-eF450 antibodies (1 µg per mouse; eBioscience). Platelets were visualized by injecting CD49b-PE antibodies (0.6 µg per mouse; BioLegend). All antibodies were injected iv via the jugular vein cannula 30 min. before imaging, except for Ly6G-AF647 antibodies, which were injected 24 h before imaging (on the second day of IMQ treatment) via the tail vein. In experiments involving adoptive transfer, antibodies (Ly6G-AF647 and CD49b-PE) were injected 30 min. before imaging, just before the injection of CellTracker-stained neutrophils. Each mouse was imaged for up to 4 h, with separate movies/images acquired during this time.

The number of neutrophils in WT and SLPI KO mice was quantified both in circulation and outside of blood vessels nearby. Cells were counted in 5 different frames of 10-min. movies acquired with IVM, with each frame separated by 2 min. of movie. At least 2 different fields of view per mouse were analyzed from *n* = 3 mice per group.

The neutrophil migration rate through the blood vessel in the psoriatic ear skin was determined throughout the course of the acquired movies. In each 10-min movie, a line of approximately 50 μm was drawn inside the blood vessel (using iQ software, Andor, Oxford Instruments). Neutrophils that had slowed down were observed, and the number of frames needed for each cell to pass the given distance was counted and later converted into real time (seconds). One 10-min movie was analyzed for each mice from *n* = 3 mice per group.

### Establishing neutrophil migration pattern in the psoriatic ear skin

The migration pattern and cell behavior in psoriatic ear skin tissue and blood vessels were analyzed using recorded movies of WT and SLPI KO mice. Each in vivo movie was opened in ImageJ (NIH) with the MTrackJ plug-in selected. For each movie, 20 cells were randomly chosen and tracked through the series of frames, marking their movement until they were no longer visible in the field of view (see supplementary Video). Each cell’s migration path was recorded in a different color, and the distance covered (in arbitrary units) was calculated automatically.

### 3D and 4D reconstruction of cross sections to analyze localization and movement of neutrophils

The number of neutrophils at different ear skin levels above the blood vessel of WT and SLPI KO mice was establish on a side view of a 3D reconstruction (IMARIS software, Bitplane, Oxford Instruments) of a series of optical cross-sections (z-stacks). A series of z-stacks through the ear skin was performed with a z-step of 1 μm approximately through 100 z-planes. Then, the ear structure was reconstructed with IMARIS software with red and violet color channels, for platelets indicating blood flow and neutrophils present in tissue for at least 24 h, respectively. Images were saved in. jpeg format and analyzed in ImageJ (NIH). An arbitrary mask, consisting of 3 frames each of approximately 17 μm length, was applied on images, and the number of neutrophils on three different levels above the blood vessel was establish.

Neutrophil migration pattern (4D) inside the ear skin was established by a time-lapse optical cross section acquisition (z-stack movies) through the mouse ear skin. The time-lapse series of optical cross sections was performed one after another for 10 min. (iQ software). Then, a 3D reconstruction in time (4D) was performed in IMARIS software (Bitplane, Oxford Instruments) and the z-stack movies were saved as. avi movie files and analyzed in ImageJ (NIH). In the latter software, 12 randomly chosen cells were followed through the time course of an entire movie and were tracked with the MTrackJ plug-in for ImageJ. A migration path was recorded for each cell and the distance (in arbitrary units) was calculated automatically. A minimum of 3 individual movies were analyzed per mouse for *n* = 3 mouse per group.

### Human neutrophils isolation and migration analysis

Human blood was collected into sodium citrate and was subjected to isolation within 1 h of draw. Neutrophils were isolated by a density gradient 1.077 g/ml centrifugation, using Pancoll (PAN Biotech) and were recovered from the corresponding erythrocyte fraction of the Pancoll gradient, as described previously [7].

To study migratory behavior, neutrophils were seeded onto microscopy plates (µ-Plate 96 well black uncoated, Ibidi) and incubated for 15 min in RPMI1640 medium supplemented with 10% FBS, or with 10% FBS and 20 µg/ml recombinant SLPI [23]. Cells were then imaged for 30min. at room temperature using time-lapse video microscopy with a motorized stage (Olympus) at a frame rate of one frame every 16s.

The generated files were analyzed, identifying migrating cells as those with elongated shapes, adherent to the surface, that displayed any movement during the observation period. Cells were counted manually and expressed as a percentage of the total cells within the fields of view (FOV).

### Preparation of human neutrophil extracts

Neutrophils isolated from the blood of healthy donors were plated on culture plates in RPMI1640 medium and incubated at 37 °C in a 5% CO_2_ atmosphere for 40 min. Recombinant SLPI [23] was added to the medium at a final concentration of 20 µg/ml, and the cells were incubated for an additional 30 min. under the same conditions. The cells were washed with PBS and centrifuged at 650 × g for 5 min. at 22 °C. The cell pellet was resuspended in relaxation buffer (10 mM PIPES, pH 7.3; 30 mM NaCl; 3.5 mM MgCl₂; 0.5 mM EGTA; 0.5 mM EDTA; and a freshly added protease inhibitor cocktail). Cells were then disrupted by cavitation in compressed nitrogen at 500 psi, centrifuged at 650 × g for 10 min. at 4 °C, and the pelleted lysates were resuspended in the relaxation buffer. Protein concentration in the samples was determined using the Bradford method. Lysates containing equal amounts of total protein were subjected to Western Blot analysis.

Recombinant SLPI was detected using primary anti-mouse His tag antibodies (Abcam) and secondary anti-mouse HRP antibodies (Abcam). A loading control was performed by detecting β-actin using primary anti-mouse β-actin antibodies (Abcam) and secondary anti-mouse HRP antibodies.

### Statistical analysis

Statistical analyses were performed using GraphPad Prism 9 (GraphPad Software). Data are shown as mean ± standard deviation (SD) or standard error of the mean (SEM). The specific tests performed and number of samples per group are described in the figure legends.

## Results

### SLPI influences neutrophil skin recruitment in an experimental model of psoriasis

**Fig. 1:**
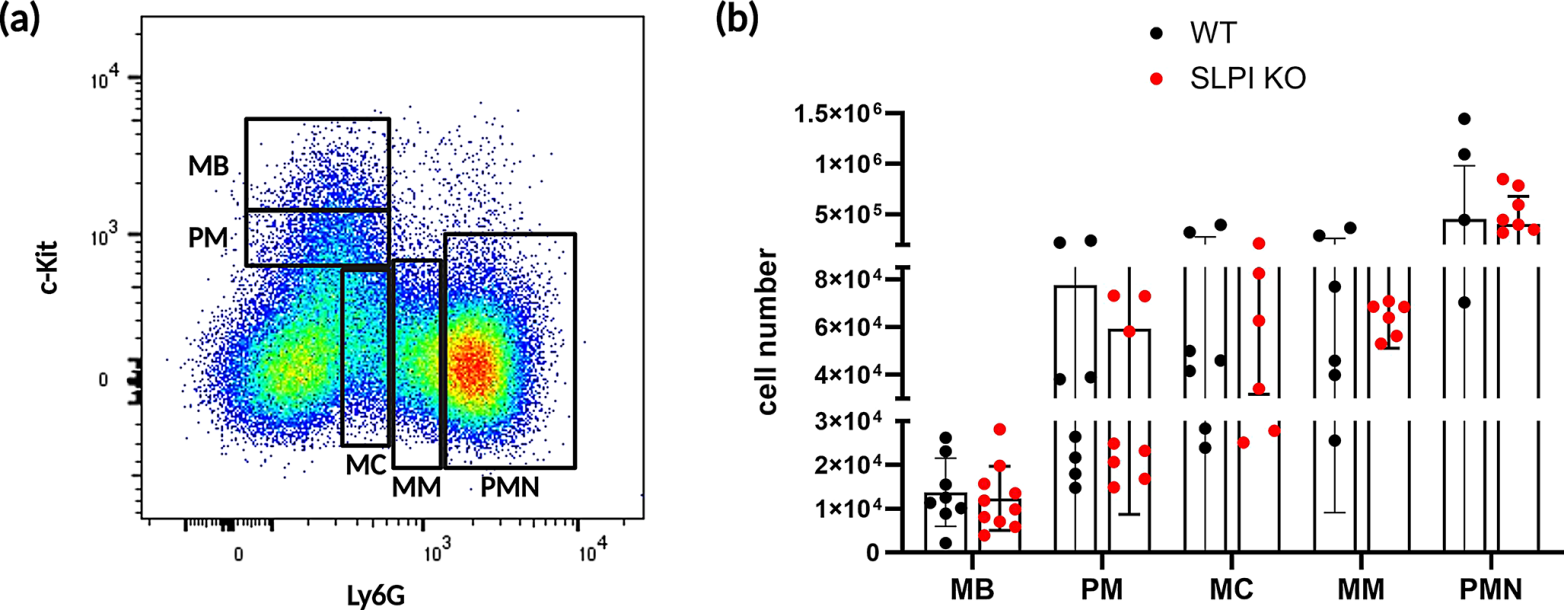
SLPI KO and WT mice share neutrophil development patterns. Linage-negative cells from the bone marrow of the indicated mice were subjected to staining for c-kit and Ly6G followed by flow cytometry analysis. (**a**) Representative flow cytometry plot from WT mice showing the gating strategy for immature and mature neutrophils. (**b**) Number of neutrophils at different stages of development. Data are shown as the mean ± SD. Dots represent individual mice. MB = myeloblasts, PM = promyelocytes, MC = myelocytes, MM = metamyelocytes, PMN = mature neutrophils

To investigate whether SLPI plays a role in neutrophil trafficking in psoriasis, we utilized SLPI knockout (KO) mice and wild-type (WT) littermate controls in an experimental imiquimod (IMQ)-based model of psoriasis-like dermatitis [19, 24]. SLPI KO and WT mice have comparable numbers of neutrophils in the bone marrow (BM) (Fig. 1). They also share similar kinetics of neutrophil development, as judged by comparable numbers of neutrophil precursor and mature neutrophil populations, including: myeloblasts, promyelocytes, myelocytes, metamyelocytes and mature neutrophils (Fig. 1a, b).

**Fig. 2:**
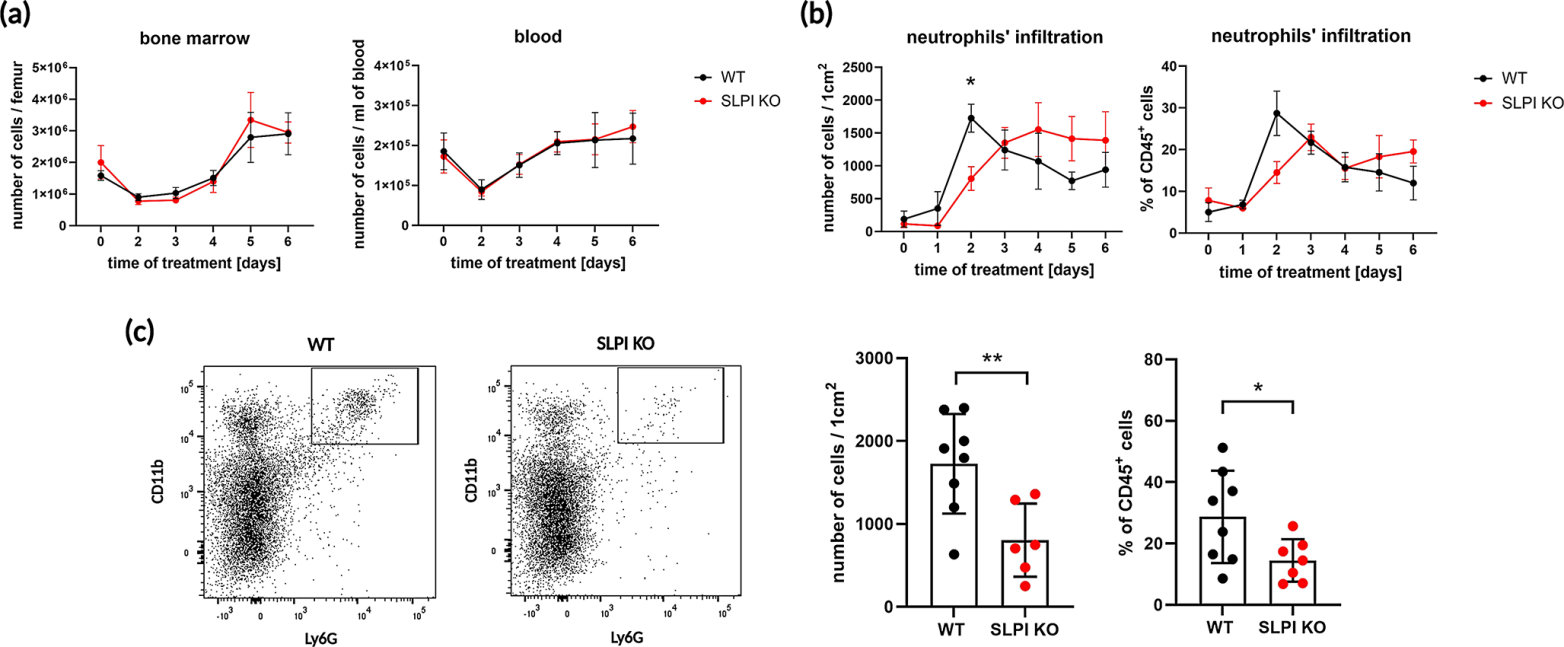
SLPI influences the kinetics of neutrophil infiltration into the skin in psoriasis-like dermatitis. The indicated mice were subjected to IMQ treatment. (**a**) Bone marrow and blood were harvested and analyzed by flow cytometry. Data are presented as mean ± SEM, with 3–6 mice per group. A mixed model ANOVA with Greenhouse-Geisser correction followed by Šídák’s multiple comparison test revealed no statistically significant differences between WT and SLPI KO mice. (**b**) Skin was harvested and analyzed by flow cytometry. Total leukocytes were detected using anti-CD45 mAbs, and neutrophils were identified using anti-Ly6G and CD11b mAbs. Data are presented as mean ± SEM, with *n* = 6–8 mice per group. Statistical significance (*p < 0.05) was determined using a mixed model ANOVA with Greenhouse-Geisser correction, followed by Šídák’s multiple comparison test. (**c**) Representative flow cytometry plots (left panel), the number of skin-infiltrating neutrophils (middle panel), and the percentage of skin-infiltrating neutrophils (right panel) at day 2 of IMQ treatment are shown. Dots represent individual mice; bars indicate the mean ± SD, *n* = 6–8 mice per group. *p < 0.05, **p < 0.01, by Student’s t-test

We administered topical treatments of IMQ-containing Aldara™ cream for up to 6 days on the ears of SLPI KO and WT mice to induce psoriatic changes [19]. Subsequently, neutrophil skin infiltration was analyzed daily in the harvested, treated skin using flow cytometry. Neutrophils numbers in the BM and the bloodstream were comparable between SLPI KO and WT mice over the whole course of experimental psoriasis (Fig. 2a). Notably, SLPI-deficient mice exhibited a diminished number and frequency of (Ly6G^+^, CD11b^+^) neutrophils among (CD45^+^) leukocytes in the skin during the early time points (Fig. 2b), with significantly fewer neutrophils present in the ear skin of SLPI KO mice compared to WT controls at day 2 (Fig. 2c). By day 3, both mouse types showed comparable numbers and percentages of neutrophils in diseased skin. However, from day 4 through day 6, a tendency for a higher accumulation of skin-infiltrating neutrophils was observed in SLPI KO mice compared to WT mice (Fig. 2b).

Taken together, although overall SLPI deficiency did not markedly influence the number of cutaneous neutrophils in this model over the 6-day treatment period, it did impact the timeline of neutrophil recruitment.

### In vivo imaging reveals SLPI-dependent differences in neutrophil extravasation

To elucidate the mechanisms underlying the delayed skin infiltration by neutrophils in SLPI-deficient mice, we conducted a spinning-disk confocal intravital microscopy (IVM) analysis of migratory neutrophils. We focused on day 2 after the IMQ challenge, which temporally aligned with the peak of neutrophil trafficking in this model.

**Fig. 3:**
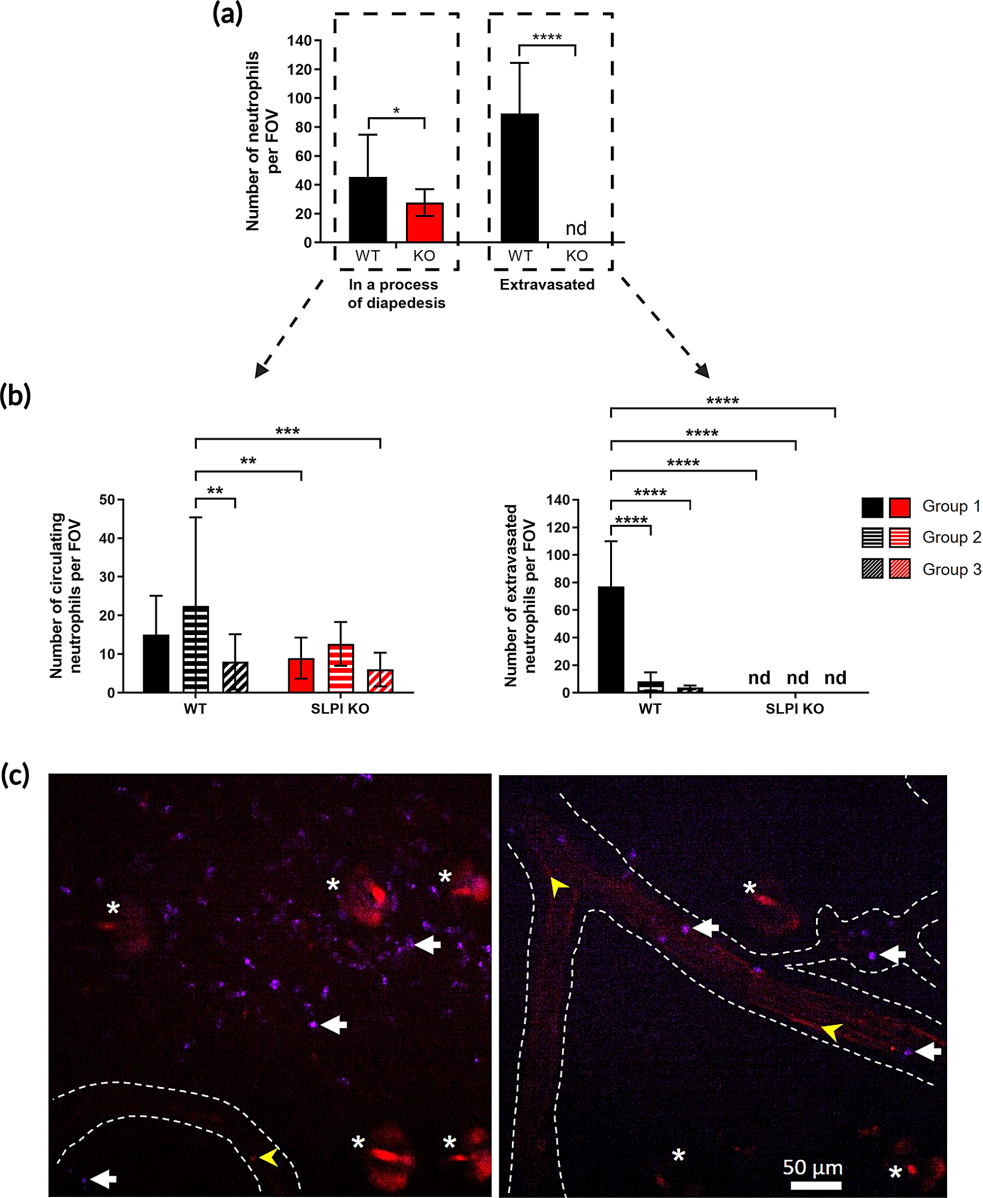
IVM visualizing psoriatic skin vasculature reveals differences in the number of vessel-interacting neutrophils between SLPI KO mice and their WT counterparts. The indicated mice were subjected to IMQ treatment for 2 days. Neutrophils were fluorescently labeled intravenously with Alexa Fluor 647-conjugated anti-mouse Ly6G 24 h before imaging, and with eFluor 450-conjugated anti-mouse Ly6G mAbs together with CD49b mAbs (to detect platelets) just before 4-hr IVM imaging. At least 2 different fields of view per mouse were analyzed. *N* = 3 mice per group. (**a**) Ly6G^+^ cells were counted in circulation (circulating neutrophils in the process of diapedesis) and also outside, but nearby, blood vessels (extravasated neutrophils). Data are presented as mean ± SD. *p < 0.05, ****p < 0.0001 by Student’s t-test. “nd"= not detected. (**b**) Neutrophils labeled 24 h before imaging (group 1), just before 4-hr imaging (group 2), or labeled by both anti-Ly6G Abs (group 3) are shown. Data are presented as mean ± SD. **p < 0.01, ***p < 0.001, ****p < 0.0001, by one-way ANOVA (Bonferroni post-hoc). “nd” indicates cells were not detected in this area. (**c**) Representative images of mouse ear skin depicting differences in the number and location of neutrophils in WT and SLPI KO mice. The blood vessel is marked with a white dotted line, exemplary neutrophils (purple) are marked with white arrows, platelets (red) that were used to indicate blood flow are marked with yellow arrowheads. Autofluorescent hair follicles are marked with asterisks. Scale bar = 50 μm

Neutrophils were fluorescently labeled at two-time points: 24 h before IVM imaging and immediately before 4-hr IVM imaging, using neutrophil-specific antibodies with distinct labels, administered i.v. This two-step staining approach before live observation of neutrophils in the bloodstream and skin tissue was chosen because only neutrophils present in circulation can be stained with i.v.-injected antibodies. Consequently, neutrophils that had already extravasated at the time of antibody administration would not be detected. We observed fewer neutrophils in total (stained by either anti-neutrophil antibodies) at various stages of diapedesis (rolling, adhering, or crawling) in SLPI KO versus WT mice (Fig. 3a, left panel), with no neutrophils identified just outside of blood vessels in SLPI KO animals (Fig. 3a, right panel).

Using this two-step staining strategy to determine the timing of neutrophil extravasation, we then focused on neutrophils stained at various time points. The first set of antibodies, applied 24 h prior to imaging, facilitated the detection of neutrophils that extravasated after this time (between 4 and 24 h prior to imaging) in the skin tissue (Fig. 3b, group 1). The second set of antibodies was applied immediately before imaging, and the fate of newly stained neutrophils was followed for the next 4 h, (Fig. 3b, group 2). While some neutrophils present in circulation were double positive, their number was negligible. These were primarily neutrophils that never extravasated and were not removed from circulation either prior to or at the time of imaging. Nevertheless, some of the double-positive neutrophils did extravasate within the last 4 h, i.e., during the time of imaging (Fig. 3b, group 3).

Considering the time spent in circulation, we found that all three populations (groups 1–3) of neutrophils underwent diapedesis in both SLPI KO and WT mice (Fig. 3b, left panel). However, predominantly neutrophils that had left circulation between 4 and 24 h prior to imaging (violet) extravasated in WT animals (Fig. 3b, right panel, group 1). Representative images are presented in Fig. 3c and supplementary video. The neutrophils that extravasated in WT mice and were visible in close proximity to the blood vessel were not stationary, and their movement could be tracked (supplementary video). In contrast, at the same distance from the blood vessel, no neutrophils were visible in the tissues of SLPI KO animals. Only neutrophils passing by in the blood could be tracked (supplementary video).

**Fig. 4:**
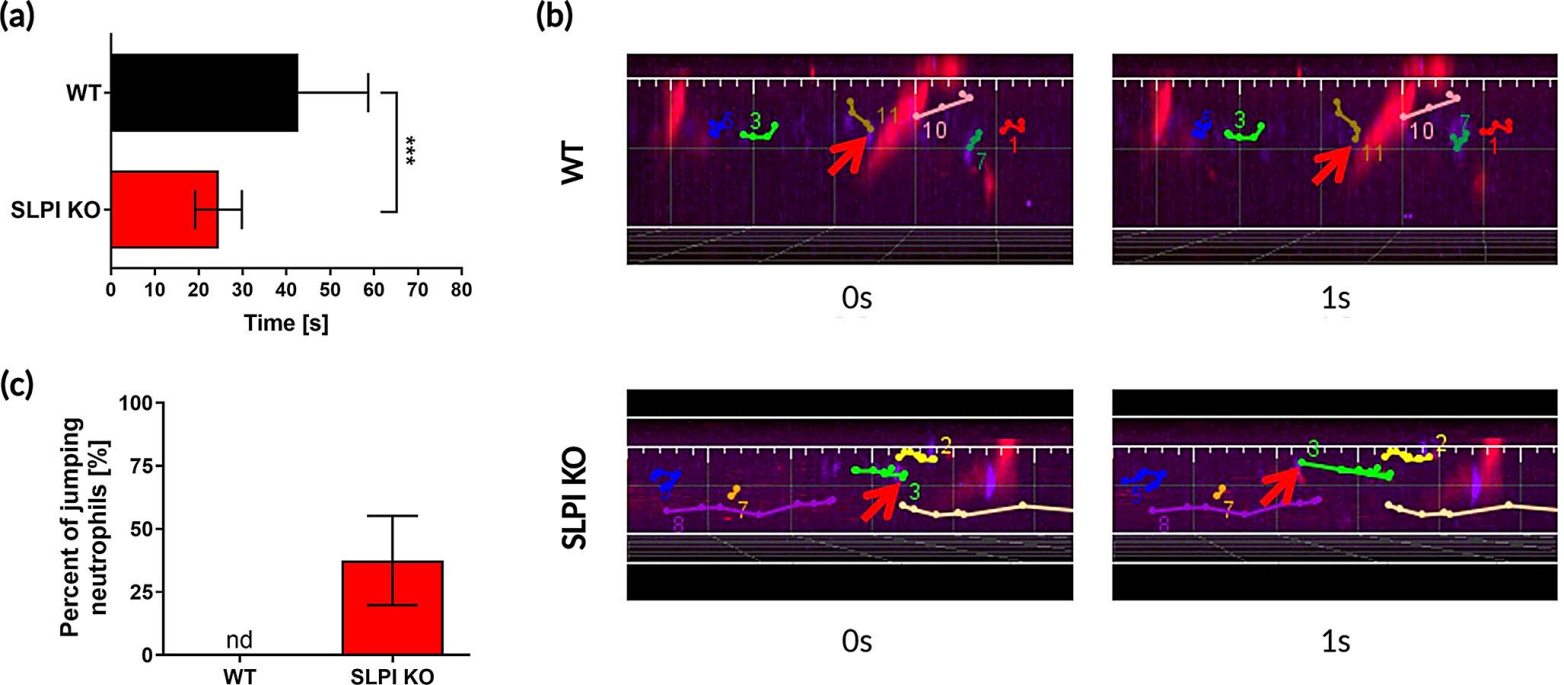
SLPI deficiency alters the neutrophil migration pattern in their interaction with vessels at the early stages of experimental psoriasis. The indicated mice were subjected to IMQ treatment for 2 days. Neutrophils were fluorescently labeled intravenously with anti-Ly6G mAbs, and platelets with anti-CD49b mAbs, followed by IVM imaging. One 10-min. movie was analyzed for each mouse. *N* = 3 mice per group. (**a**) Time in seconds needed for neutrophils to travel a distance of 50 μm inside the ear skin blood vessel. Data are presented as mean ± SD, ***p < 0.001 by Student’s t-test. (**b**) Representative images of mouse ear skin capturing the motion of neutrophils within the blood vessels. Points indicate the current location of a neutrophil in a chosen timeframe, while white lines depict the path traveled. Exemplary neutrophil trajectories are shown, with the red arrow indicating the current cell position. (**c**) Percentage of neutrophils executing “jumps” within the blood vessels. Data are presented as mean ± SD. “nd” =not detected

While fewer neutrophils were observed performing diapedesis in the SLPI KO mice (Fig. 3a), SLPI KO neutrophils exhibited higher speeds in the bloodstream (requiring less time to pass the same distance) than WT cells (Fig. 4a), suggesting earlier stages of rolling.

When monitoring single neutrophils in blood of SLPI KO and WT mice in 1s frames, we observed a fraction of neutrophils exhibiting “jumping-like” behavior that resulted in covering much larger distances over equivalent time frames (Fig. 4b). This “jumping-like” movement was detected only in SLPI-deficient mice, where approximately 37.5% of neutrophils were found to behave this way (Fig. 4c).

Together, these findings suggest that “jumps” reflect markedly altered ability of neutrophils to firmly interact with endothelial cells in the absence of SLPI.

### Extravasated SLPI KO and WT neutrophils differ in their movement speed and localization pattern within psoriatic skin

**Fig. 5:**
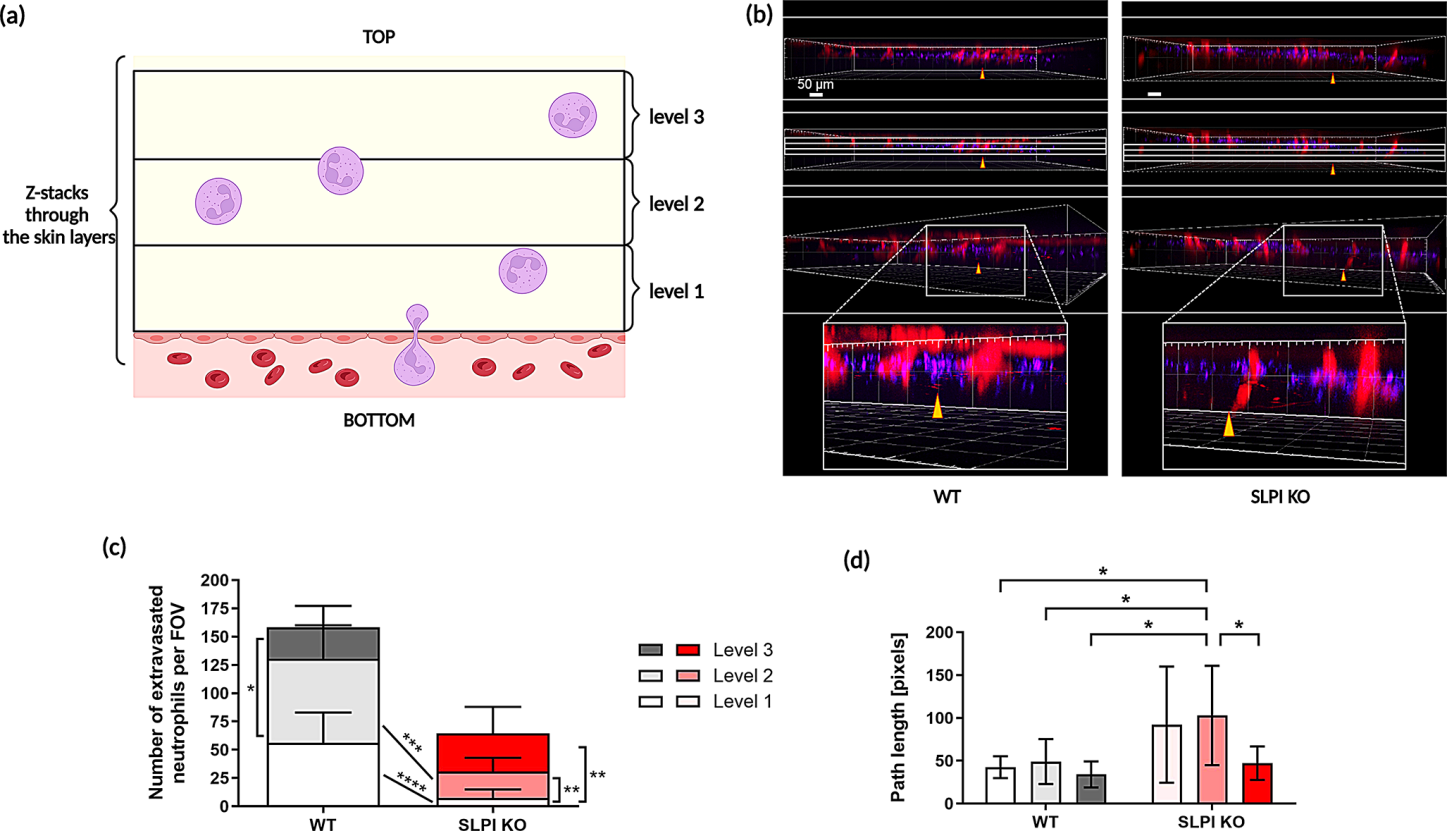
In SLPI-deficient mice, there are more extravasated neutrophils far from the vessel compared to WT mice. The indicated mice were subjected to IMQ treatment for 2 days. Neutrophils were fluorescently labeled intravenously with anti-Ly6G mAbs, and platelets with anti-CD49b mAbs, followed by IVM imaging. (**a**) Schematic of the counting strategy: A series of optical cross-sections (z-stacks) were made through the ear skin. An arbitrary mask was applied to estimate the number of neutrophils at three different levels above the blood vessel, where level 1 is the closest to the vessel and level 3 is the closest to the skin surface. (**b**) Exemplary images of z-stacks made through the skin of SLPI KO mice and their WT counterparts. A blood vessel (marked with a yellow arrowhead) is identified by platelet flow (red). Neutrophils are stained in violet. (**c**) Extravasated neutrophils were counted in arbitrary compartments above the blood vessel. Data are present as mean ± SD * *p* < 0.05, ** *p* < 0.01, *** *p* < 0.001, **** *p* < 0. 0001 by Student’s *t*-test. A minimum of 3 intravital movies were analyzed per mouse, *n* = 2 mice per group. (**d**) Cell movement: absolute path length of neutrophils at different levels of psoriatic ear skin tissue above the blood vessel from which they extravasated in SLPI KO and WT animals. Data are presented as mean ± SD. * *p* < 0.05 by one-way ANOVA (Bonferroni post-hoc)

Flow cytometry of IMQ-treated ears revealed an accumulation of neutrophils in the skin of either SLPI KO or WT mice by day 2, albeit to a different extent (Fig. 2b, c). Since at this time we did not detect any neutrophils outside blood vessels in SLPI KO mice by IVM imaging, which covered a small skin area in close proximity to the blood vessel (Fig. 3a), next, we analyzed neutrophil movement following neutrophil extravasation from the blood by IVM imaging a larger skin area. We applied a mask on the 3D reconstruction of z-stacks denoting three different levels of the skin in which the z-stacks were acquired (Fig. 5a, b). Each level was approximately 17 ± 2 μm thick. Overall, fewer neutrophils were detected outside of the vasculature in SLPI KO mice. However, most of the extravasated cells were found in the outermost analyzed area (level 3), closest to the skin surface (Fig. 5c). The extravasated SLPI KO neutrophils moved (level 2) or tended to move (level 1) longer distances when located closer to the blood vessels, whereas they showed less mobility when located closer to the skin surface (level 3) (Fig. 5d).

Together, these findings suggest that the lack of SLPI causes neutrophils to migrate further away from the vasculature, traveling longer distances in the skin and positioning themselves closer to the skin surface.

### Deficiency of SLPI in neutrophils partly explains the differing abilities of neutrophil extravasation in SLPI KO mice

The altered neutrophil migration in SLPI-deficient mice could result from the absence of SLPI in the neutrophils themselves or in other cells within the neutrophil’s microenvironment.

To assess the intrinsic impact of SLPI on neutrophil migration, we employed an adoptive transfer approach. Specifically, untreated WT and SLPI KO BM-derived neutrophils were labeled with distinct cell trackers. These donor WT and SLPI KO neutrophils were then intravenously administered in equal numbers into recipient WT mice and, in parallel, into SLPI KO mice that had undergone IMQ treatment for 2 days. To distinguish exogenous from endogenous neutrophils, recipient mice were intravenously stained with fluorescently labeled anti-Ly6G mAbs immediately before the donor neutrophil injection and the 4-hr IVM imaging. Endogenous neutrophils were Ly6G^+^ but negative for the CellTracker signal, unlike the exogenous cells.

**Fig. 6:**
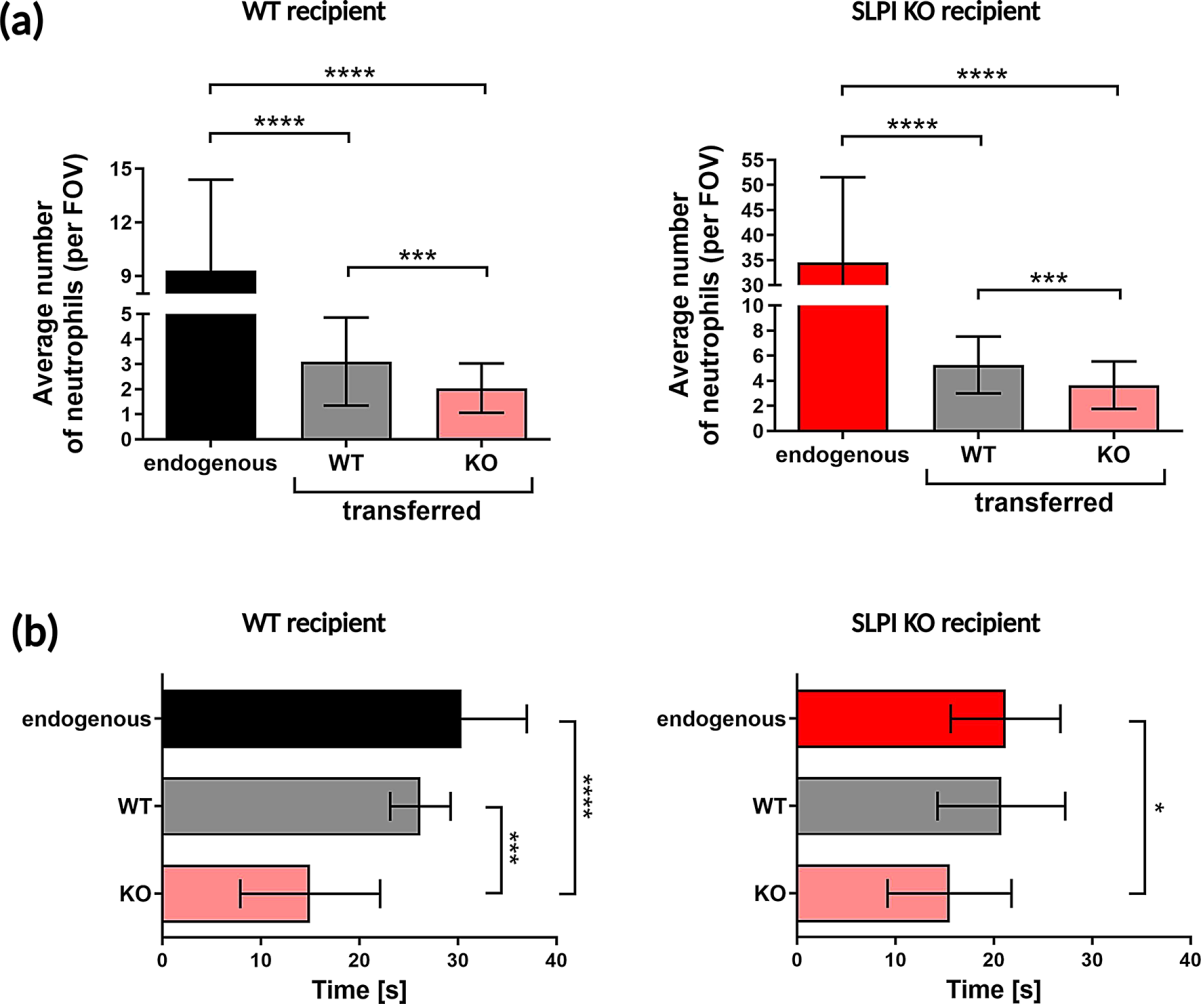
Adoptively transferred neutrophils from WT and SLPI KO mice differ in their extravasation potential. The indicated recipient mice were subjected to IMQ treatment for 2 days. Donor neutrophils were isolated from the bone marrow of untreated WT and SLPI KO mice, fluorescently labeled with different cell trackers, and administered in equal amounts to the recipient mice. Neutrophils in the recipient mice were labeled with anti-Ly6G mAbs followed by 4-hour IVM imaging of ear skin blood vessels. (**a**) The number of endogenous neutrophils, exogenous WT neutrophils, and exogenous SLPI KO neutrophils in WT recipients (left panel) and SLPI KO recipients (right panel) are shown. The data are presented as mean ± SD of at least three different fields of view (*n* = 2 mice per group), *** *p* < 0.001, **** *p* < 0.0001 by one-way ANOVA (Bonferroni post-hoc). (**b**) The time required for endogenous neutrophils and neutrophils transferred from WT and SLPI KO donor mice to traverse a distance of 50 μm within the blood vessels of WT recipients (left panel) and SLPI KO recipients (right panel). The data are shown as mean ± SD. * *p* < 0.05, *** *p* < 0.001, **** *p* < 0.0001 by one-way ANOVA (Bonferroni post-hoc)

In both WT and SLPI KO recipient mice, the blood vessels of the psoriatic ear skin were dominated by endogenous neutrophils, which were the most noticeable in the field of view (Fig. 6a). Unexpectedly, the number of endogenous neutrophils undergoing diapedesis was higher in the recipient SLPI KO mice compared to WT mice (Fig. 6a, right and left panels, respectively). This increase in diapedesis in SLPI KO mice might be attributed to a higher number of extravasation-capable neutrophils newly released from the bone marrow into the blood in response to the adoptive transfer.

However, when we directly compared the adoptively transferred neutrophils in recipient WT mice, significantly more exogenous WT neutrophils underwent extravasation compared to SLPI-deficient neutrophils (Fig. 6a, left panel). In similar experiments where SLPI KO mice served as recipients, we again observed more donor WT than SLPI KO neutrophils interacting with the endothelial beds in the ear skin blood vessels (Fig. 6a, right panel).

Taken together, these data suggest that SLPI-deficient neutrophils have a reduced ability to extravasate, regardless of the presence or absence of SLPI in other cells along the routes of neutrophil diapedesis.

We next tracked the time taken by both endogenous and exogenous neutrophils from WT and SLPI KO mice to travel the same distance in the blood following neutrophil transfer. Under adoptive transfer conditions, endogenous neutrophils in WT mice were slower (took longer time to travel the distance) compared to endogenous neutrophils in SLPI KO mice (Fig. 6b, right and left panel, respectively). SLPI KO neutrophils, when administered together with WT neutrophils to WT mice, required the least time to travel this distance and showed a similar tendency when SLPI KO mice were the recipients (Fig. 6b).

Taken together, these data suggest a higher speed of movement for SLPI-deficient neutrophils in the bloodstream and, consequently, reduced interaction of these neutrophils with the vessel wall in psoriatic skin.

### SLPI effect on migration of human neutrophils

**Fig. 7:**
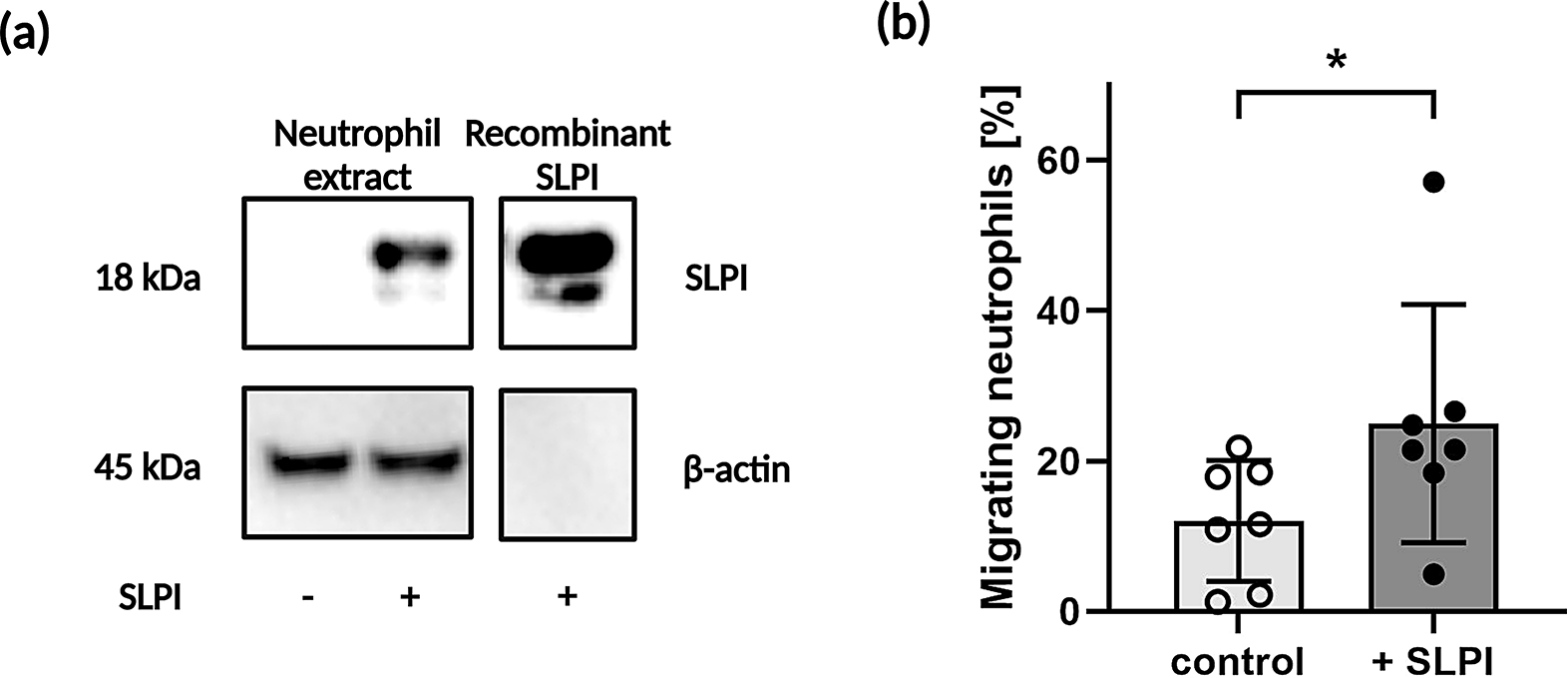
Exogenous, cell-penetrant SLPI enhances human neutrophil migration ability. Neutrophils were isolated from the blood of healthy donors. (**a**) Neutrophils were incubated with 20 µg/ml recombinant SLPI for 30 min or left untreated. Neutrophil lysates were then subjected to Western blot analysis (left panel). A control of 50 ng recombinant SLPI is shown (right panel). (**b**) Neutrophils were incubated with media supplemented with 10% FCS (control) or media supplemented with 10% FCS and 20 µg/ml SLPI for 15 min, followed by 30-min. migration assays. Time-lapse video microscopy showed the percentage of migratory cells in total cells per field of view (FOV), *n* = 7. *p < 0.05; Student’s *t-*test

To determine whether SLPI affects the migration of human neutrophils, we treated neutrophils isolated from the blood of healthy human donors with neutrophil-penetrant recombinant SLPI (Fig. 7a). This was followed by time-lapse videomicroscopy (Fig. 7b). Although only approximately 10% of neutrophils underwent migration after seeding in tissue culture dishes, a significantly increased fraction of adherent and actively migrating cells was observed following a 15-min. pretreatment and 30 min. of neutrophil imaging in the presence of SLPI (Fig. 7b).

Taken together, these data support a pro-migratory role of SLPI.

## Discussion

Here, we demonstrate that SLPI controls the dynamics of neutrophil skin infiltration under conditions of chronic inflammation. The absence of SLPI leads to aberrant neutrophil migratory behavior, resulting in significantly delayed infiltration of psoriatic skin during the early stages of skin lesion development and distribution of dermal neutrophils closer to the skin surface. Similarly, human neutrophils exhibit better migration in the presence of recombinant SLPI, suggesting that SLPI supports the migratory behavior of neutrophils across species.

Although the exact mechanism by which SLPI mediates this response is yet to be determined, SLPI is primarily recognized as an inhibitor of serine proteases, such as NE and CatG. Considering these functions, SLPI deficiency in neutrophils could affect their ability to extravasate by disrupting protease activity regulation. For example, excessive NE in the cytosol, unchecked by SLPI, could degrade F-actin [14], thereby reducing the ability of neutrophils to interact with endothelial cells and migrate out of the vasculature at the early stages of psoriasis. Furthermore, the reduced capacity of SLPI-deficient neutrophils to interact with the vessel wall during the early stages of psoriasis might result from the degradation of trafficking-supporting factors, such as specific adhesion molecules and chemoattractants. These factors could be targeted by serine proteases, including NE and CatG, which are no longer regulated by SLPI. This aligns with the known degradative properties of NE and CatG against various chemokines and adhesion molecules, including ICAM-1 and VCAM-1 [25, 26].

However, other chemoattractants and adhesion molecules required for breaching the vessel wall (such as IL-8 and JAM-C, respectively) may be more resistant to cleavage by SLPI-controlled serine proteases or may even require processing by these proteases to promote neutrophil migration. For instance, the levels of endothelial junction molecule JAM-C are reduced through NE-mediated cleavage, and this reduction coincides with heightened neutrophil transmigration [13]. Scenarios where SLPI-regulated serine protease activity contributes to more efficient neutrophil migration may characterize the later stages of neutrophil recruitment in psoriasis. This is supported by our observation that, during the later stages, the absence of SLPI no longer impairs neutrophil recruitment to the skin and may even enhance it (Fig. 2).

It is also possible that, at later stages, neutrophils released from the BM of SLPI KO mice exhibit a distinct adhesive phenotype, enhancing their ability to interact with the vessel wall compared to neutrophils from WT mice. Consequently, the impaired migration of neutrophils observed in the absence of SLPI during the early stages of psoriasis might be compensated for at later stages by the release of neutrophils that no longer rely on SLPI function for transmigration. This hypothesis is supported by observations indicating that SLPI plays a role in regulating neutrophil release from the BM.

Neutrophils begin expressing SLPI at the early stages of their maturation in the BM [27]. The SLPI protein is stored in the secondary granules of mature, neutrophils released into the bloodstream, but it can also be detected in the cytosol and nucleus of these cells [18, 23]. SLPI protein levels are upregulated in the skin of psoriasis patients compared to healthy individuals, with keratinocytes and neutrophils serving as notable sources of this protein in psoriatic skin [8, 19].

Circulating neutrophils exhibit differences in SLPI protein levels and its intracellular distribution [8], suggesting that SLPI function is regulated at several levels in these cells. Thus, it is likely that at least some neutrophils employ SLPI to facilitate their rapid egress from the vasculature and recruitment into developing psoriatic skin lesions.

What would be the relevance of differential SLPI-dependent recruitment of neutrophils to chronically inflamed skin? Given the postulated pro-inflammatory and debris-clearing functions of neutrophils, the suppression of early stages of neutrophil skin infiltration in the absence of functional SLPI might have significant pathophysiological consequences for psoriasis. On one hand, SLPI shortage in neutrophils might postpone neutrophil-mediated inflammatory responses in the skin. On the other hand, slower mobilization of neutrophils due to a lack of SLPI function might delay the clearing of tissue debris by neutrophils. As a result, the accumulation of DAMPs in the skin would be expected, thereby exacerbating inflammation.

Since SLPI not only impacts the timeline of neutrophil skin recruitment but also their tissue distribution, the final outcome of SLPI-mediated cutaneous presence of neutrophils might be skin-region dependent. After crossing the vessel wall, neutrophils move toward the skin surface, but they are also observed accumulating close to the vessels. Our IVM data demonstrate that at early stages of psoriasis development, neutrophils in SLPI KO mice exhibit “jumping behavior” in their interaction with the endothelium. After crossing the vessel wall, they distribute further away from the vasculature compared to WT neutrophils and ultimately migrate a longer distance into the dermis. These findings suggest that the downregulation or inaccessibility of SLPI could result in neutrophils scanning a larger area of activated endothelium before breaching the vascular wall. In the extravascular setting, the distribution of neutrophils closer to the skin surface enables these cells to be more engaged in the upper dermis regions, where they are more likely to encounter bacterial or epidermal components. On the other hand, SLPI-sufficient neutrophils would localize in close proximity to their skin entry vessels, potentially contributing to vascular remodeling and dilation.

Since SLPI is found not only in neutrophils but also in other cells, such as endothelial cells and platelets (human protein atlas [28]), it may regulate neutrophils’ migratory behavior intrinsically or with assistance from other cells within the neutrophils’ microenvironment, including the BM and blood vessels. The former may control the differentiation stage at which neutrophils are released into the blood, while the latter may regulate the process of crossing the vascular barrier at sites of skin inflammation.

In our adoptive transfer experiments, we employed BM-derived neutrophils. WT and SLPI KO mice have similar BM neutrophil repertoires (Fig. 1). Using BM-derived neutrophils instead of blood-derived neutrophils allowed us to bypass potential differential recruitment from the WT and SLPI KO BM, as both WT and SLPI KO mice had a mixed population of neutrophils that could otherwise be differentially mobilized in an SLPI-dependent manner. The adoptively transferred BM WT and SLPI-deficient neutrophils demonstrated a differential ability to interact with the vessel wall. This was evidenced by either a significantly reduced number of SLPI KO neutrophils performing diapedesis or a higher movement speed within vessels, indicating a weaker or earlier-stage interaction with the vessel wall.

The BM-derived, adoptively transferred WT and SLPI KO neutrophils replicated the migratory behavior of endogenous circulating neutrophils in WT and SLPI KO mice during the early stages of psoriasis (Fig. 6 vs. Fig. 3). Based on these findings, we conclude that SLPI supports neutrophil extravasation at this initial phase of skin lesion development, acting at least partly as an inherent neutrophil regulator.

However, in the adoptive transfer settings, the behavior of recipient SLPI KO neutrophils differed from that of transferred SLPI KO neutrophils. This was evident when comparing the time it took for both endogenous and exogenous neutrophils in SLPI KO mice to travel a 50 μm distance in the blood. Unlike exogenous and endogenous WT neutrophils in WT mice, which moved at similar speeds, endogenous neutrophils in SLPI KO mice were slower than transferred SLPI KO neutrophils (Fig. 6b). Additionally, after adoptive transfer, endogenous SLPI KO neutrophils showed a higher propensity for extravasation compared to endogenous WT neutrophils, whereas the opposite was observed in WT and SLPI KO mice without additional neutrophils (Fig. 3 vs. Figure 6).

A plausible explanation for these findings is that newly released neutrophils in SLPI KO and WT mice, mobilized by disruptive cues in the BM, differ in their ability to extravasate to chronically inflamed skin tissue. The adoptive transfer of neutrophils likely prompts a rearrangement of the BM niche in recipient mice. This could lead to alterations in the release of neutrophils into the bloodstream, such as the premature mobilization of not fully developed neutrophils into the circulation.

## Conclusions

Our studies support a model in which SLPI-dependent neutrophil migration to psoriatic skin is regulated at multiple levels:

1. In the Bone Marrow: While SLPI is not essential for neutrophil development, it regulates the spatiotemporal kinetics of neutrophil mobilization into the bloodstream. During the early stages of psoriasis, neutrophils in WT mice are more efficient than those in SLPI KO mice. However, at later stages, neutrophils in SLPI KO mice tend to outperform their WT counterparts. This disparity may result from differences in the rolling and adhesive properties of prematurely released neutrophils, rather than direct effects of SLPI itself.
2. In the Circulation: SLPI functional sufficiency enhances neutrophil extravasation and recruitment to psoriatic skin during the early stages of the disease.
3. In Extravascular Cutaneous Sites: In the presence of SLPI, neutrophils initially localize near skin entry vessels in the dermis, increasing their interaction with perivascular constituents and antigens in deeper skin layers. This positioning may contribute to vascular dilation. In contrast, the absence of SLPI causes neutrophils to accumulate closer to dermal sites near the skin surface, exposing them to microbial stimuli or components from damaged keratinocytes. This exposure may exacerbate inflammation in the upper layers of the skin.

## Electronic supplementary material

Below is the link to the electronic supplementary material.

Supplementary Material 1

Supplementary Material 2

## Data Availability

All data related to this paper are presented in figures and in supplementary video.

## Abbreviations

BM: Bone marrow
IMQ: Imiquimod
IVM: Intavital microscopy
i.v.: Intravenously
KO: SLPI knockout
NE: Neutrophil elastase
SLPI: Secretory leukocyte protease inhibitor
WT: Wild type
