# Supplementary material for: SLPI controls neutrophil migration abilities and impacts neutrophil skin infiltration in experimental psoriasis
Source: Cell Mol Life Sci. 2025 Feb 10;82(1):74. doi: 10.1007/s00018-025-05606-y (PMC11810868; doi:10.1007/s00018-025-05606-y)
Supplement: Supplementary file 2 — Supplementary Material 2 [file 18_2025_5606_MOESM2_ESM.docx]

**Supplementary information**

**Video:** Exemplary movies of psoriatic ear skin of WT and SLPI KO mice, related to Fig. 3.
